# i3Drefine Software for Protein 3D Structure Refinement and Its Assessment in CASP10

**DOI:** 10.1371/journal.pone.0069648

**Published:** 2013-07-19

**Authors:** Debswapna Bhattacharya, Jianlin Cheng

**Affiliations:** 1 Department of Computer Science, University of Missouri, Columbia, Missouri, United States of America; 2 Department of Computer Science, Informatics Institute, Bond Life Science Center, University of Missouri, Columbia, Missouri, United States of America; University of Alberta, Canada

## Abstract

Protein structure refinement refers to the process of improving the qualities of protein structures during structure modeling processes to bring them closer to their native states. Structure refinement has been drawing increasing attention in the community-wide Critical Assessment of techniques for Protein Structure prediction (CASP) experiments since its addition in 8^th^ CASP experiment. During the 9^th^ and recently concluded 10^th^ CASP experiments, a consistent growth in number of refinement targets and participating groups has been witnessed. Yet, protein structure refinement still remains a largely unsolved problem with majority of participating groups in CASP refinement category failed to consistently improve the quality of structures issued for refinement. In order to alleviate this need, we developed a completely automated and computationally efficient protein 3D structure refinement method, i3Drefine, based on an iterative and highly convergent energy minimization algorithm with a powerful all-atom composite physics and knowledge-based force fields and hydrogen bonding (HB) network optimization technique. In the recent community-wide blind experiment, CASP10, i3Drefine (as ‘MULTICOM-CONSTRUCT’) was ranked as the best method in the server section as per the official assessment of CASP10 experiment. Here we provide the community with free access to i3Drefine software and systematically analyse the performance of i3Drefine in strict blind mode on the refinement targets issued in CASP10 refinement category and compare with other state-of-the-art refinement methods participating in CASP10. Our analysis demonstrates that i3Drefine is only fully-automated server participating in CASP10 exhibiting consistent improvement over the initial structures in both global and local structural quality metrics. Executable version of i3Drefine is freely available at http://protein.rnet.missouri.edu/i3drefine/.

## Introduction

The biennial community-wide Critical Assessment of protein Structure Prediction (CASP) experiment aims to evaluate the progress and challenges in the state-of-the-art of protein structure modeling techniques, one of the fundamental problems in computational biology- prediction of the tertiary structure of protein from its sequence information. During the recent CASP experiments, encouraging and consistent progress have witnessed in template-based modeling (TBM) [1]–[4] or ab-initio (free-modeling; FM) [5]–[8] folding of protein structures. The refinement category has been a recent addition to the CASP framework since CASP8, which aims to evaluate whether further improvement is possible to the best predictions made by contemporary structure prediction techniques. In the blind refinement experiment, predictors are given a starting structure evaluated by the organizers as the best submitted model during the structure prediction phase (TS category) along with the sequence information. Occasionally, some hints are also provided to aid the refinement like the focus regions during refinement or the accuracy of the starting structure.

Since its inclusion during CASP8, refinement category has been drawing increasing attention by the community. During recently concluded CASP10 refinement experiment, a 92% increase in the number of refinement targets and 39% increase in the number of participating groups have been observed compared to CASP9. This is not unexpected because a consistent and efficient refinement protocol can serve as a natural end step in almost all the contemporary structure prediction pipelines adding value to the already predicted structures through simultaneous improvement in backbone geometry and correction of local errors like irregular hydrogen bonding, steric clashes, unphysical bond length, unrealistic bond angles, torsion angles and side-chain χ angles. However, structure refinement has proven to extremely challenging as revealed in the assessment of refinement experiments during CASP8 and CASP9 [9], [10] with only a few participating groups were able to improve the model quality consistently. It should be noted, however, that CASP refinement category differs in a slight but significant way from refinement in the context of TBM [11]–[19] where the objective is to refine the best identified template structure(s) to produce better quality prediction. In CASP, on the other hand, the starting models issued for refinement have already been refined by other structure prediction pipelines and judged to be the best among all the submitted models. Thus, attempts to improve qualities of these models would naturally impose more challenges and often the risk of degrading the model quality instead of improving it.

In view of the major difficulties in the field, we developed a consistent and computationally efficient refinement algorithm, called 3Drefine [20] by optimizing the hydrogen bonding network and atomic level energy minimization using a composite physics and knowledge-based force field. We participated in CASP10 refinement category with an iterative version of 3Drefine protocol, i3Drefine. As per the official CASP10 results released during CASP10 meeting in the form of assessors’ presentation (http://predictioncenter.org/casp10/docs.cgi?view=presentations), i3Drefine was ranked as the single best refinement server method capable of consistent improvement in qualities of starting structures. The contribution of this article are two-fold: (1) Providing the community with access to a fast, accurate and freely downloadable executable version of refinement software which could be used to improve the qualities of the models coming from variety of protein structure prediction methods, or to act as the end-game strategy in a TBM pipeline and (2) evaluation of its performance in CASP10 refinement experiment to analyse the effectiveness of this method in a strict blind mode. Although CASP10 refinement category includes both human and server predictors, since i3Drefine is a fully automated server, this article will be mainly focused on the assessment of refinement in the context of automated server predictions.

## Materials and Methods

### i3Drefine Algorithm

i3Drefine is an iterative implementation of the energy minimization technique, 3Drefine for protein structure refinement. The details of 3Drefine protocol has been described in [20]. Here, we present a brief overview of 3Drefine algorithm.

3Drefine refinement protocol involves a two-step process: (1) Optimizing hydrogen bonding network and (2) atomic-level energy minimization using a combination of physics and knowledge based force fields; implemented using the molecular modeling package MESHI [21]. Given a starting structure for refinement, a combination of local geometry restraint and a conformational search is first performed in order to optimize the hydrogen bonding network. The optimized structure is called extended atomic model. Subsequently, 200,000 steps of energy minimization is employed on the extended atomic model using highly convergent limited memory Broyden–Fletcher–Goldfarb–Shannon (L-BFGS) [22] algorithm or until convergence to machine precision using a customized all-atom force field. The force field consists of a combination of physics based and knowledge based terms. The energetic contributions of the bonded interactions described in ENCAD potential [23] (bond length, bond angle, and torsion angle) along with tethering term of the C_α_ and C_β_ atoms [20] constitute the physics-based part while atomic pairwise potential of mean force [24] and explicit hydrogen bonding potential [25] account for the knowledge-based terms. A detailed analysis of the relative importance of these energy terms has been presented in the published work of 3Drefine [20]. The energy-minimized model is the refined model.

In i3Drefine, we use an iterative version of 3Drefine method. In order to escape from the local minima and move closer to the native structure, the starting model is minimized using 3Drefine protocol and the resulting refined model is again processed by the same method. This iteration is done five times to generate five refined models for the starting structure. Because 3Drefine invokes restrained backbone flexibility during energy minimization due to the inclusion of the knowledge-based terms in the all-atom force field, such an iterative scheme is effective. Furthermore, because of the computationally inexpensive nature of 3Drefine protocol, this iterative strategy does not provide significant computational overhead in i3Drefine pipeline consuming only a few minutes (typically less than 15 minutes) to generate five refined structures at a 2.4 GHz CPU.

### Programming Language, Platform and External Programs

The core of i3Drefine is developed in Java (http://www.java.com/en/) on top of MESHI [21] software package and the command-line interface to perform the refinement is developed in Perl programming language (http://www.perl.org/). For a seamless installation and usage of i3Drefine, a Java version 6.0 or above and Perl version 5.8.8 or above is recommended. Also, since some of the energy terms in the customized force fields require the secondary structure assignment of the starting structure for accurate calculations, DSSP program [26] needs to be used in conjunction with i3Drefine. The detailed installation instructions along with typical example of using i3Drefine have been provided in the user manual file supplied with the software. i3Drefine has been tested on 64-bit Linux based platform. However, because of the platform independent nature of Java and versatile platform support of Perl, it can be fairly easily modified to run for Windows or Mac OSX platforms.

### Metrics used for Evaluation

We evaluate the quality of the structural refinement using both global and local measures. We focus on GDT-TS [27] and RMSD [28] score to measure of the global positioning of C_α_ atoms. Global distance cutoff sidechain (GDC-SC) [2] has been used as a global quality metric for sidechain positioning. To assess the local qualities of the models, we use MolProbity score [29] as a local measure of physical correctness of a structure and SphereGrinder [10] as a local all-atom measure of structural similarity. Finally we use a recently introduced contact area difference (CAD) score [30] which quantifies the differences between physical contacts in the models before and after refinement with respect to their native structures.

### GDT-TS

GDT-TS [27] is a global quality measure of the correct positioning of backbone based on multiple superpositions of the predicted and experimental structure. It counts the average percentage of residues with C_α_ atom distance from the native structure residues below 1, 2, 4, and 8 Å, respectively, after optimal structure superposition. GDT-TS ranges from [0, 1] with higher value indicating better accuracy.

### RMSD

Similar to GDT-TS, RMSD [28] is a global measure of the correct positioning of the C_α_ atoms. However, RMSD is based on a single superposition lacking any kind of distance cutoffs. Hence, RMSD and GDT-TS is weekly correlated. Furthermore, unlike GDT-TS, a lower RMSD value indicates that the predicted structure is close to its native state.

### GDC-SC

GDC-SC [2] has been used as a global quality metric for sidechain positioning. Unlike GDT-TS, which is focused on C_α_ atoms, GDC-SC use a single characteristic atom near the end of each sidechain. Also, 10 different superpositions with different weighting schemes are employed to calculate GDC-SC.

### MolProbity

In order to evaluate the physical realism and the local errors, we use MolProbity [29] – a single and composite score to measure local model quality. The MolProbity score denotes the expected resolution of the protein model with respect to standard experimental structures and therefore, lower MolProbity score indicates more physically realistic model.

### SphereGrinder

We use SphereGrinder to measures the local environment around each residue which was used in the refinement assessment of CASP9 [10]. SphereGrinder is based on an all-atom RMSD fit between the experimental and predicted structures using a sphere constructed by considering the set of atoms within 6 Å of the C_α_ atoms for each residue in experimental structure.

### CAD-AA

CAD score [30] is a newly introduced quality metric which is based on contact area difference between predicted and experimental structure, thereby directly reflecting interactions within the protein structure. The contact area is calculated based on a protein structure tessellation approach [31] and normalized between [0, 1] with higher value indicating better structure. We use the all-atom version of the CAD score, namely, CAD-AA.

### Normalizing and Overall Quality Score

Higher value of GDT-TS, GDC-SC, SphereGrinder and CAD-AA scores indicate better models while lower values RMSD and MolProbity scores represent better models. In order to effectively compare the degree of refinement between different groups or targets, a single overall quality score is essential. We use a robust version of Z-score based on median absolute difference (MAD) of the changes in quality of the models induced through refinement. This is a slightly modified approach used in refinement assessment during CASP9 [10].

The difference in the model quality is first calculated to get the delta quality score for a given quality metric (e.g. GDT-TS).
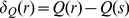
(1)where Q(r) and Q(s) denote the quality score for refined and starting structures respectively corresponding to quality measure Q.

For a given target, we calculate the MAD using:
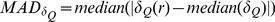
(2)where median(δ_Q_) denotes the median of the delta score for the corresponding quality metric and |.| is the absolute value. The robust Z-score is then calculated as:



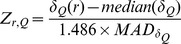
(3)The factor 1.486 scales the MAD to be same as standard deviation of a normal distribution.

Finally, a weighted average of Z-score is taken for all different quality metrics to combine the results of all six scores into a single score, called Q-score.
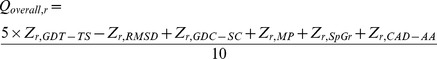
(4)


In this scoring scheme, GDT-TS is given a weight of 5, which makes half of the overall score and other five metrics makes the other half. Although this procedure is arbitrary, it emphasizes the improvement in backbone positioning as judged by GDT-TS score, a widely used metric by CASP assessors, compared to other measures.

## Results and Discussion

The fully automated i3Drefine software was first blindly tested in CASP10 refinement experiment, 2012 with the group name MULTICOM-CONSTRUCT (Server group 222). Since then, we systematically evaluate its performance using global and local quality metrics like GDT-TS, RMSD, GDC-SC, MolProbity, SphereGrinder and CAD-score and perform comparative analysis of i3Drefine against all the groups participating in CASP10 refinement category. Here, we first summarize the targets offered for refinement during CASP10 refinement experiment along with the measures of the initial quality. Secondly, we present the automated server groups participating in CASP10 refinement category and introduce a pseudo group called “Void” as a control. Thirdly, we assess the overall degree of refinement produced by i3Drefine in a strict blind mode. Fourthly, a comparison of i3Drefine against the state-of-the-art refinement server methods participating in CASP10 has been presented along with head-to-head comparison of the scores and their statistical significance. During CASP10, each predictor was asked to submit up to five predictions while ranking submissions from best to worst. We, therefore, perform one set of analysis using the first submitted model, which is the best prediction as per the ranking from the predictor. However, because predictors often fail to correctly rank their submissions, we present a second set of analysis by selecting the best prediction (as evaluated by our overall quality score) from each group for each target. The comparison between the first and the best predicted models by i3Drefine also reveals the advantages of the iterative version of our refinement method (i3Drefine) over the non-iterative version (3Drefine). Finally, we compare i3Drefine with the top five non-server (human) methods and discuss the added benefits of human predictors and the possibility of adopting them in computational structure prediction pipelines.

### Targets used for Refinement in CASP10

Table 1 summarizes the targets issued for refinement in CASP10 and the measures of the initial quality of these targets. The occasional “hints” provided by the organizers to focus on certain segment(s) of the structures during refinement has also been reported. These are the starting models for refinement and were chosen from the top submissions during the structure prediction category. These models, therefore, represent one of the best predicted structures submitted by the community for each target and intuitively, consistent refinement of these structures is a nontrivial task.

**Table 1:** Summary of CASP10 refinement targets.

#	Target	Residues	Method	GDT-TS	RMSD (Å)	GDC-SC	MP^a^	SG^b^	CAD-AA	Focus^c^
1	TR644	141	X-ray	0.8422	2.712	0.4346	2.49	0.7518	0.69	–
2	TR655	175	NMR	0.6871	4.654	0.2853	3.83	0.5143	0.58	6–20; 51–64
3	TR661	185	X-ray	0.800	2.743	0.375	1.11	0.7135	0.67	–
4	TR662	75	NMR	0.8267	2.031	0.3364	2.42	0.7600	0.67	–
5	TR663	152	X-ray	0.6908	3.372	0.2626	4.05	0.7697	0.67	53–78; 141–181
6	TR671	88	X-ray	0.5568	7.716	0.1158	3.68	0.4432	0.59	–
7	TR674	132	X-ray	0.8523	3.444	0.4417	2.99	0.7424	0.69	–
8	TR679	199	X-ray	0.7186	3.949	0.3076	1.15	0.5226	0.6	25–45; 146–156; 187–197
9	TR681	191	X-ray	0.7827	2.273	0.3274	2.89	0.6387	0.64	–
10	TR688	185	X-ray	0.7838	2.524	0.4249	1.77	0.7730	0.67	–
11	TR689	214	X-ray	0.8773	1.660	0.4202	3.18	0.8738	0.72	–
12	TR696	100	X-ray	0.7075	3.519	0.2631	2.97	0.5000	0.58	–
13	TR698	119	X-ray	0.6471	4.653	0.2568	2.73	0.6555	0.63	17–35; 90–100
14	TR699	225	X-ray	0.8411	2.211	0.3361	2.77	0.7733	0.66	–
15	TR704	235	X-ray	0.6989	2.78	0.2325	2.89	0.7319	0.64	–
16	TR705	96	X-ray	0.6458	4.709	0.2211	3.63	0.3750	0.52	–
17	TR708	196	X-ray	0.8648	4.630	0.4551	2.65	0.8214	0.71	–
18	TR710	194	X-ray	0.7487	2.440	0.3628	0.50	0.7732	0.72	–
19	TR712	186	X-ray	0.9261	1.992	0.5515	2.69	0.8817	0.77	80–89; 116–129; 141–155
20	TR720	198	X-ray	0.5783	8.515	0.2558	1.33	0.4697	0.58	–
21	TR722	127	X-ray	0.5709	4.422	0.1614	0.88	0.8976	0.72	–
22	TR723	131	X-ray	0.8511	2.232	0.3772	2.21	0.8473	0.68	–
23	TR738	249	X-ray	0.9006	1.396	0.5036	2.38	0.9398	0.75	17–35; 90–100
24	TR747	90	X-ray	0.825	1.956	0.3796	1.95	0.6778	0.63	–
25	TR750	182	X-ray	0.7679	2.125	0.348	2.49	0.7967	0.67	–
26	TR752	148	X-ray	0.9037	1.495	0.4305	1.52	0.7973	0.71	41–50; 100–110; 125–128
27	TR754	68	NMR	0.7794	2.410	0.1997	2.56	0.8235	0.65	–

a MolProbity scores of the starting structures.

b SphereGrinder scores of the starting structures.

c The numbers indicate the range of focus residues as suggested by CASP10 organizers.

### Server Groups Participating in CASP10 Refinement Category

A total of fifty groups participated in CASP10 refinement experiment including both human and server predictors. Thirteen groups took part as fully automated server predictors. The server predictors were given a three days deadline to submit the refined structures to the prediction centre as opposed to a three weeks deadline offered for the human predictors. In Table 2, we summarize the server groups participating in CASP10 along with the number of predictions submitted by each predictor. The performance of fully automated i3Drefine method (group name MULTICOM-CONSTRUCT) can be directly compared to these methods on the CASP10 refinement targets. This would enable us to assess the ability of i3Drefine protocol with state-of-the-art automated refinement methods in a strict blind mode. Groups attempting more that 50% of the targets have been highlighted in bold in Table 2.

**Table 2:** List of server groups participating in CASP10 refinement category.

#	Group #	Group Name^a^	Targets attempted	Total submitted models	First submitted model^b^
1	006	**MUFOLD-QA**	22	110	22
2	028	**YASARA**	18	18	18
3	103	PconsM	2	10	2
4	108	**PMS**	27	135	27
5	124	PconsD	2	10	2
6	175	**FRESS_server**	27	135	27
7	179	Lenserver	2	10	2
8	198	**chuo-fams-server**	27	27	27
9	222	**MULTICOM-CONSTRUCT** c	27	135	27
10	238	**chuo-repack-server**	26	26	26
11	286	Mufold-MD	1	5	1
12	292	Pcons-net	2	10	2
13	424	**MULTICOM-NOVEL**	27	135	27

a Group name in bold indicates the group has attempted more than 50% of refinement targets.

b Models submitted with a Model ID of one.

c CASP10 group name for i3Drefine.

As a control, we created a pseudo group called “Void” group. This group represents the starting model provided by the CASP organizers for refinement. We judge the success and degree of refinement with respect to the ‘Void’ group. Groups that perform worse than Void group have on average degraded the quality of starting structures rather than improving it.

### Overall Performance of i3Drefine in CASP10 Blind Refinement Experiment

**Figure 1:**
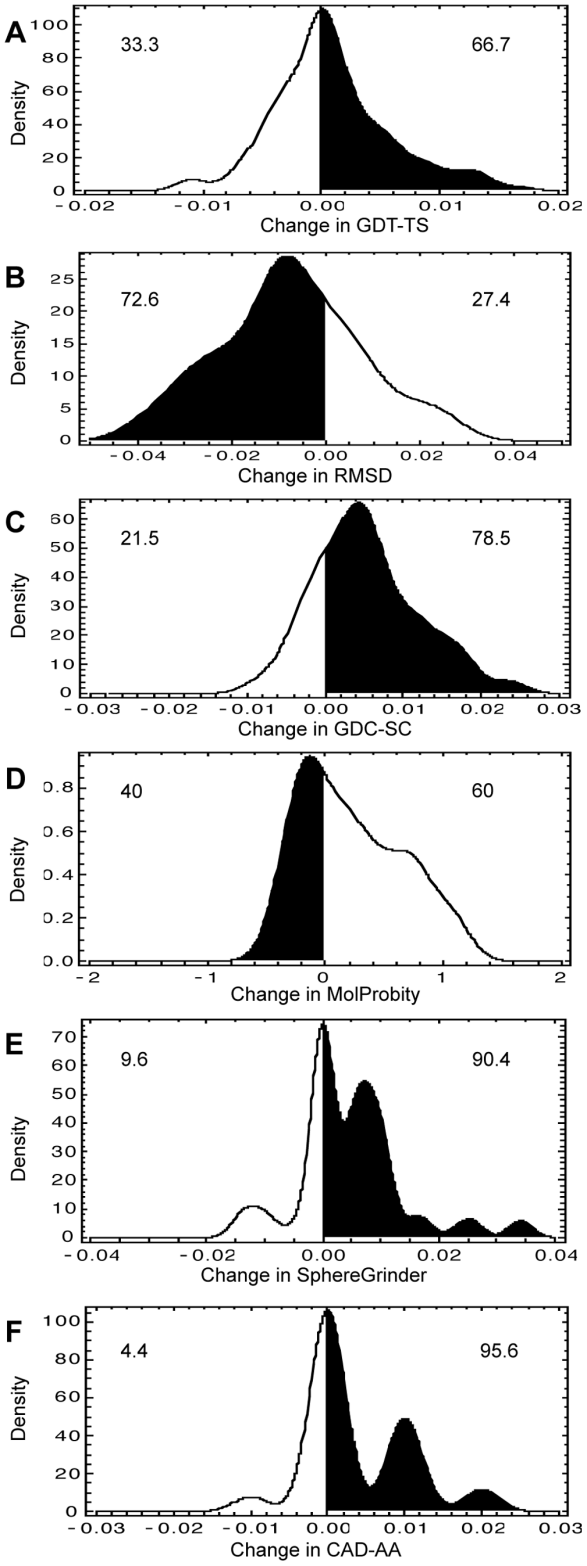
Distribution of i3Drefine refinement for all submitted structures. Distributions of change in quality scores after i3Drefine refinement are shown for these metrics: (A) GDT-TS, (B) RMSD, (C) GDC-SC, (D) MolProbity, (E) SphereGrinder and (F) CAD-AA. Regions shaded in black indicate improvement over the starting model. The numeric values are the percentage of times the structures were made better or worse for each metric.

Figure 1 shows the distribution of change in model quality relative to the starting model as judged by the score difference in six quality metrics for all submitted model by i3Drefine method for all CASP10 refinement targets. Positive changes in GDT-TS, GDC-SC, SphereGrinder and CAD-AA scores represent refinement success whereas negative changes in RMSD and MolProbity scores indicate a failure in refinement. In Figure 1, the regions shaded in black indicate improvement in the corresponding quality measure with the numbers above these regions representing the percentage of refinement successes while the regions without shading indicate degradation in the model quality and the numbers specify the percentage of failures in refinement. While for most metrics, the number of improvements significantly outnumbered number of failures, the improvement is typically modest in nature. For example, refinement successes outnumber failures by more than a factor of three in global position of the backbone atoms as judged by GDT-TS and RMSD scores and global quality of sidechain positioning as measured by GDC-SC score. While most of ΔGDT-TS, ΔRMSD and ΔGDC-SC scores lie within ∼ ±4%, the distributions are skewed towards improvement. Highly consistent improvement has also been observed in the local quality measures like ΔSphereGrinder and ΔCAD-AA scores and the distributions are highly skewed towards success with over 90% success. However, for MolProbity score, there are more failures than success and the distribution is marginally skewed towards failure. The distributions in Figure 1 are multimodal, which indicate that not all targets are equally easy to refine and the degree of refinement vary with the difficulty of targets.

**Figure 2:**
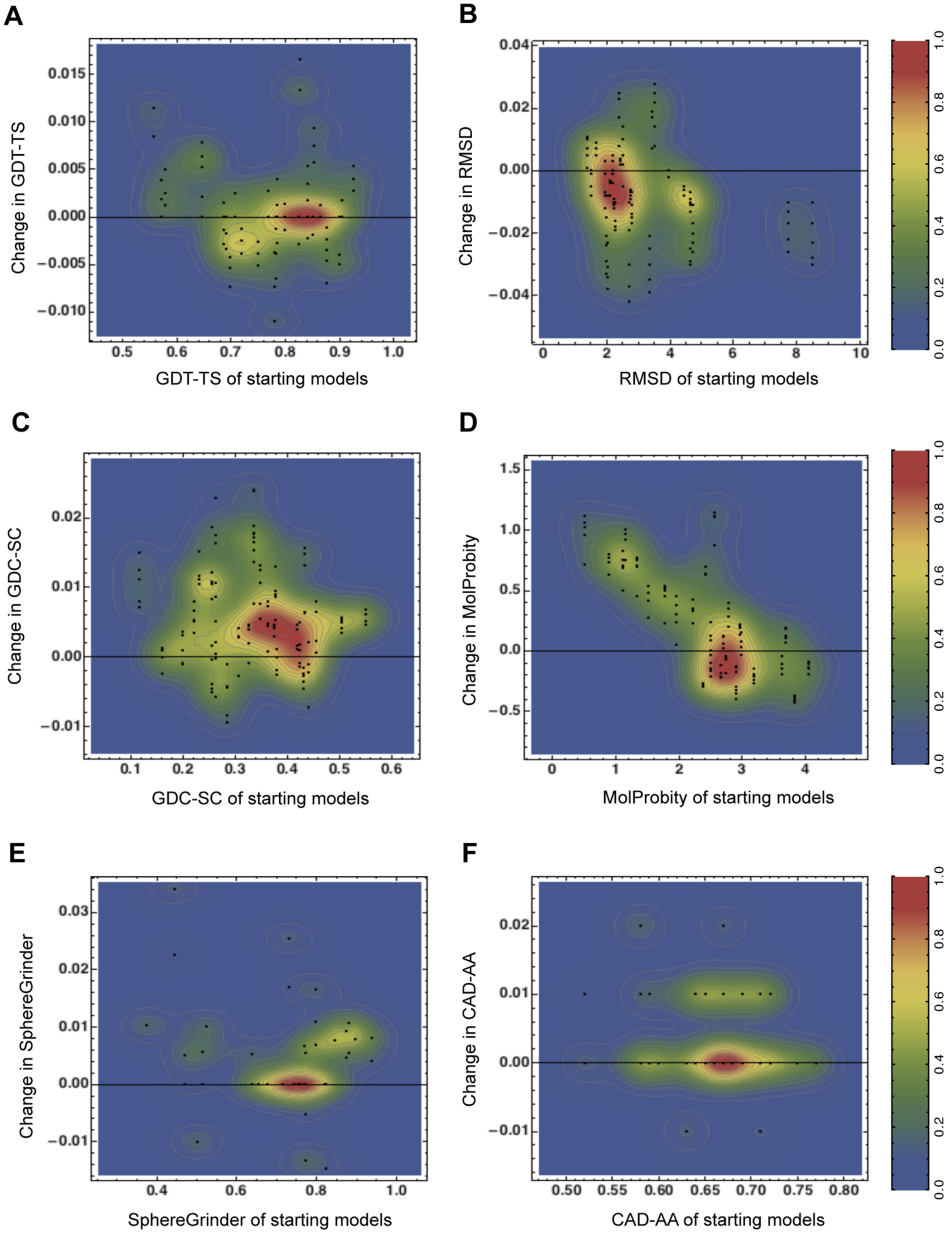
Distributions of score changes with respect to the quality of starting structures. Relationships between changes in quality scores and the quality of the starting models are shown for these metrics: (A) GDT-TS, (B) RMSD, (C) GDC-SC, (D) MolProbity, (E) SphereGrinder and (F) CAD-AA. The Black points indicate the actual data points while the contours are filled with colours that vary from blue for low density to red for high density. The colour function has been scaled between 0 and 1 and the legends are shown on the right.

In Fig. 2, we examine the relationship between the starting score of any of the quality measures and the ability of i3Drefine to improve the starting model. Although, it is difficult to infer a conclusive correlation between them with only 27 targets, some interesting trends can be observed. For example, most of the starting structures have quite accurate backbone positioning with only 7 out of 27 targets have RMSD score more than 4Å and GDT-TS less than 0.7. For these moderate-accuracy targets, i3Drefine always improves the backbone quality by increasing GDT-TS score and reducing RMSD score. For the more accurate starting structures with RMSD ∼ 2Å, the RMSD distribution is skewed towards improvement. However, there are approximately as many improvements as failures in GDT-TS score for high-accuracy targets (GDT-TS more than 0.8). The global quality of sidechains, as measured by GDC-SC varies from 0.1 to 0.6 indicating that the starting structure set comprises a wide variety in terms of accuracy of sidechain positioning, although most of the targets are in the range of 0.3 to 0.5. Promisingly, i3Drefine consistently improves the GDC-SC score irrespective of the quality of starting structures. When the initial model has less accurate local quality as measured by MolProbity (MolProbity score is more than 2), we observe consistent improvement in MolProbity. However, i3Drefine almost always increases MolProbity score indicating degradation in local model quality when MolProbity score is less than 2. For other local quality measures like SphereGrinder and CAD-AA, we observe a modest but consistent improvement in the model quality across all target difficulty. In short, more consistent and simultaneous improvements both in global and local quality measures have been observed for moderately accurate targets than high-accuracy targets.

**Figure 3:**
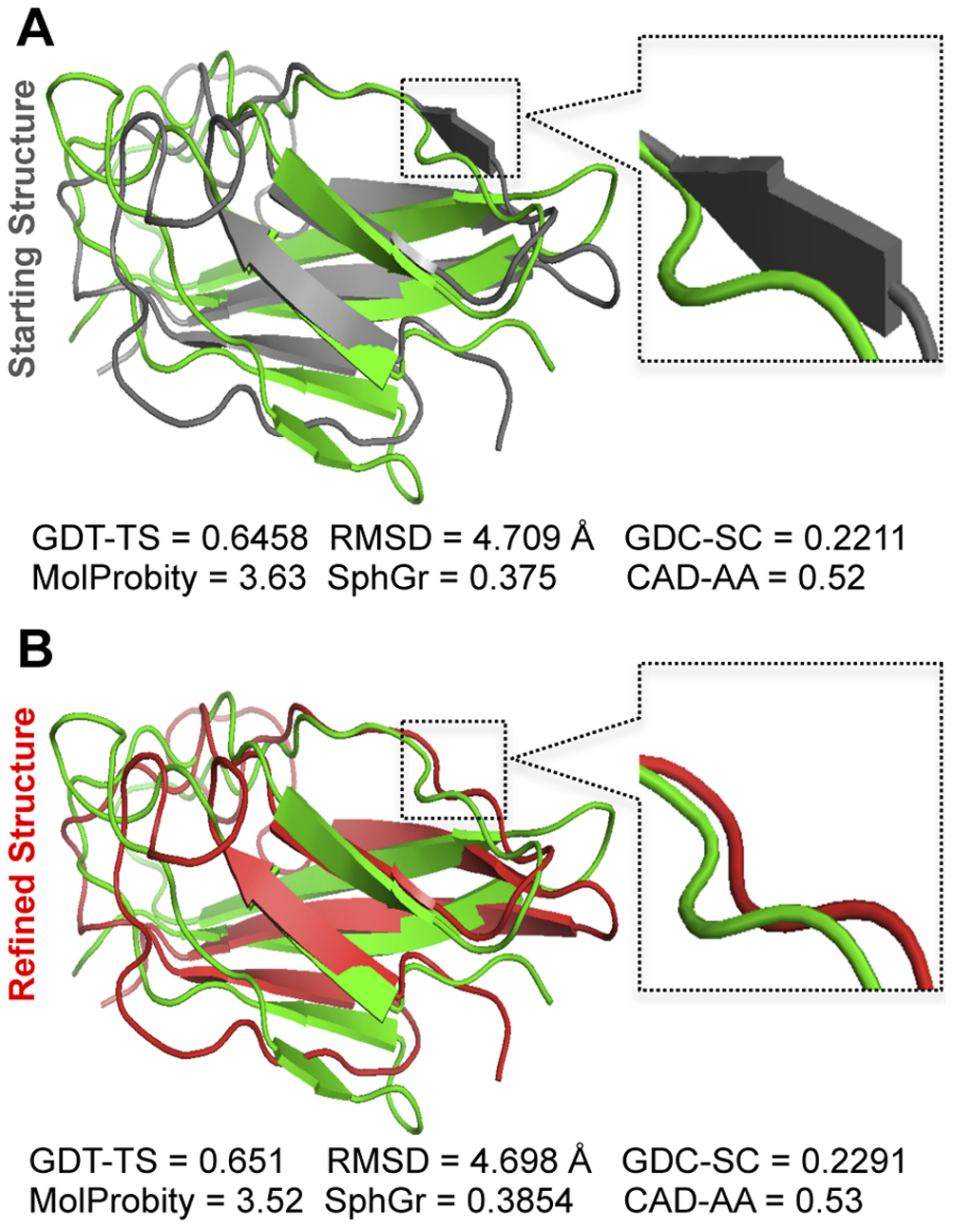
Example of i3Drefine refinement for CASP10 target TR705. (A) Structural superposition of initial model (grey) on native structure (green). (B) Structural superposition of refined model using i3Drefine (red) on native structure (green). The values of the quality measures before and after refinement have been reported under the models. The black dotted square highlights the region with prominent structural improvements and a closer look of the change is shown in the right.

A representative example of refinement has been presented in Fig. 3 for CASP10 refinement target TR705. i3Drefine refinement results in GST-TS, GDC-SC, SphereGrinder and CAD-AA scores to increase from 0.6458, 0.2211, 0.375 and 0.52 to 0.651, 0.2291, 0.3854 and 0.53 respectively. The RMSD and MolProbity score decreases from 4.709 Å and 3.53 to 4.698 Å to 3.52 respectively. Clearly, a modest yet consistent improvement in all quality measures has been observed. More pronounced structural improvement in terms of backbone positioning has been observed around residue 58 where a disoriented strand region is rearranged to a coil, thereby bringing the refined model closer to the native state.

### Comparison of i3Drefine with other Server Predictors Participating in CASP10

We compare the performance of i3Drefine with the thirteen server predictors participating in CASP10 refinement category based on the first submitted model and the best submitted model as judged by our overall quality score, Q_overall_. It can be noticed form Table 2 that some of the predictors attempted very few targets and only eight groups (including i3Drefine) submitted prediction for more than 50% of targets (i.e. more than 13 targets). Although we have taken into account all the submitted models by every group while performing our analysis, we choose to focus on these eight predictors for a fair comparison between them. To compare predictors with a single score, we have computed the sum of Q_overall_ for each predictor and ranked groups based on that.

**Figure 4:**
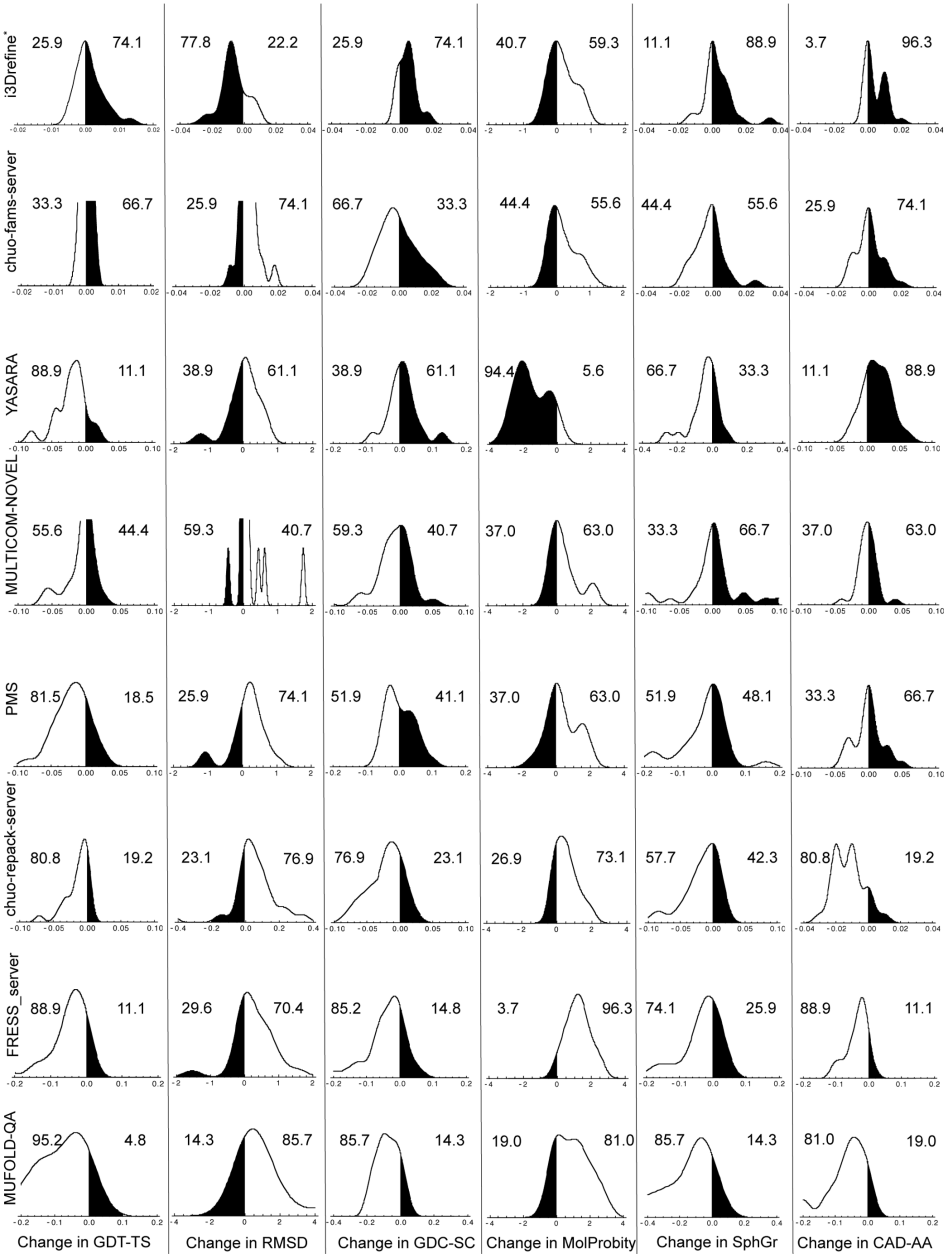
Distribution and degree of refinement for top server groups based on first submitted model. Distribution and degree of score changes relative to starting models for the 8 groups based on the first submitted models. The X-axis shows changes in scores with respect to the starting model. Regions shaded in black indicate improvement over the starting model. The numeric values are the percentage of times the structures were made better or worse than the starting model for each metric. The groups are ordered by the sum of overall quality score. * CASP10 group name for i3Drefine is MULTICOM-CONSTRUCT.

Upper part of Table 3 summarizes cumulative change in all the quality measures with respect to the starting structures (represented as ‘Void’ group) for eight server predictors. The groups have been ordered based on the cumulative Q_overall_ score for all the submitted targets. The results demonstrate except MolProbity score, i3Drefine improves all the quality measures in terms of cumulative change with respect to the starting structures. In Figure 4, we present the distributions of changes in model quality relative to the starting models for the eight server predictors as measured by six quality metrics. Similar to Figure 1, the regions shaded in black in Figure 4 correspond to refinement successes while the regions without shading indicate failures in refinement. We also report the percentage of successes and failures for each quality measures. The distributions for each predictor are multimodal due to variations in the quality of the starting models. Also, the degree of change in the quality score varies between predictors and type of quality metric. We, therefore, choose to maximally cover the range of score changes for each predictor and each quality measure. Some interesting variations between groups can be observed and often a trade-off exists between the extent of improvement and consistency. For example, groups like i3Drefine and chuo-fams-server perform modest but consistent improvement in almost all the scores. While the delta scores for these predictors usually lie within ∼ ±4%, the distributions are skewed towards improvement. On the other hand, there exist more adventurous groups like YASARA and MULTICOM_NOVEL capable of performing larger improvements at the cost of consistency. Also, different server predictors excel at different aspects of refinement. For instance, i3Drefine improves GDT-TS and RMSD, GDC-SC, SphereGrinder and CAD-AA scores more frequently than any other groups. The ability of YASARA to improve the GDC-SC, MolProbity and CAD-AA scores in terms of degree of change and consistency is quite impressive. The most striking feature we observe is the inability of predictors to improve the backbone positioning as judged by GDT-TS and RMSD scores. i3Drefine is the only server method able to perform consistent improvement in backbone quality as measured by a simultaneous improvement in ΔGDT-TS and ΔRMSD scores. Clearly, most of the predictors are better at improving general physicality of the starting structures than at improving the backbone positioning.

**Table 3:** Cumulative improvement relative to starting model for the top server groups in CASP10 refinement experiment.*

Selection	Group Name	ΔGDT-TS	ΔRMSD (Å)	ΔGDC-SC	ΔMP^a^	ΔSG^b^	ΔCAD-AA	Q_overall^c^
First Model	MULTICOM-CONSTRUCT^d^	0.036	−0.165	0.120	3.93	0.096	0.120	12.69
	Void	0.000	0.000	0.000	0.00	0.000	0.000	10.91
	chuo-fams-server	0.003	0.074	−0.191	5.05	−0.803	−0.060	5.71
	YASARA	−0.372	−2.138	0.188	−27.75	−0.800	0.270	2.79
	MULTICOM-NOVEL	−0.490	27.479	−0.294	11.72	−0.253	−0.090	−0.97
	PMS	−0.534	9.606	0.097	9.73	−0.624	−0.010	−5.97
	chuo-repack-server	−0.364	1.939	−0.523	13.86	−0.439	−0.430	−8.27
	FRESS_server	−1.574	10.155	−1.102	32.79	−1.512	−0.930	−39.53
	MUFOLD-QA	−2.194	140.087	−1.604	20.37	−3.121	−1.340	−109.18
Best Model	MULTICOM-CONSTRUCT^d^	0.068	−0.224	0.151	4.25	0.124	0.140	13.96
	PMS	−0.162	−2.091	0.382	8.29	−0.025	0.170	13.72
	Void	0.000	0.000	0.000	0.00	0.000	0.000	10.91
	MULTICOM-NOVEL	−0.220	14.010	−0.105	9.79	−0.059	0.010	7.49
	chuo-fams-server	0.003	0.074	−0.191	5.05	−0.803	−0.060	5.71
	YASARA	−0.372	−2.138	0.188	−27.75	−0.800	0.270	2.79
	chuo-repack-server	−0.364	1.939	−0.523	13.86	−0.439	−0.430	−8.27
	FRESS_server	−0.926	4.787	−0.648	30.10	−0.865	−0.520	−21.75
	MUFOLD-QA	−1.968	31.832	−1.289	13.17	−2.436	−0.980	−62.30

* The values for all quality metrics represent the cumulative change relative to the starting structures for all targets.

a Cumulative change in MolProbity score.

b Cumulative change in SphereGrinder score.

c Sum of overall quality score for all targets.

d CASP10 group name for i3Drefine.

**Figure 5:**
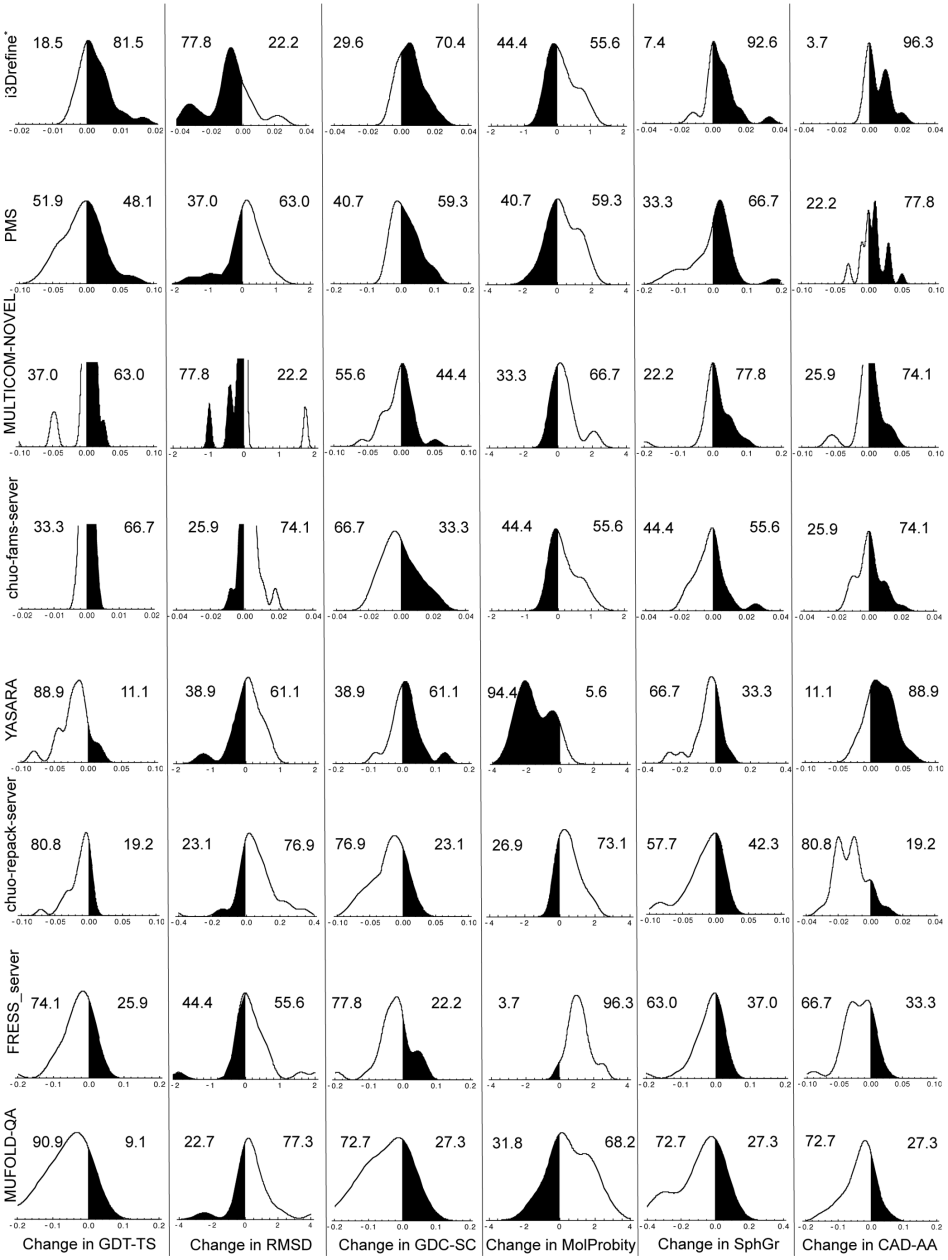
Distribution and degree of refinement for top server groups based on best submitted model. Distribution and degree of score changes relative to starting models for the 8 groups based on the best submitted models as judged by quality score for each target. The X-axis shows changes in scores with respect to the starting model. Regions shaded in black indicate improvement over the starting model. The numeric values are the percentage of times the structures were made better or worse than the starting model for each metric. The groups are ordered by the sum of overall quality score. * CASP10 group name for i3Drefine is MULTICOM-CONSTRUCT.

Because the predictors often face difficulty in correctly ranking their submissions, the first models are often not the best submitted one. To overcome this challenge, we have recalculated the results by examining only the best structure for each group (as judged by Q_overall_) for each target. In case the groups (like YASARA, chuo-fams-server and chuo-repack-server) submitted only one model as prediction, we are left with the only choice to select that as best prediction. When judged by their best model for each target, there are two groups that perform better than the ‘Void’ pseudo group as shown in the lower part of Table 3. Once again, i3Drefine outperform all the server predictors with a consistent improvement in all the quality measures except MolProbity as evaluated by cumulative change in scores. The only other group that perform better than ‘Void’ is PMS with an impressive ability to improve the overall RMSD, GDC-SC and CAD-AA scores. We observe a consistent improvement in cumulative scores changes for the predictors submitting multiple predictions when the best models are selected from each group. The distributions of changes in model quality relative to the starting models are captured in Figure 5 with the best submitted model for each predictor as judged by six quality metrics. Once more, we see a clear trade-off between consistency and degree of refinement and different predictors performing well at different aspects of refinement. It can be observed from Figure 5, that i3Drefine improves GDT-TS and RMSD, GDC-SC, SphereGrinder and CAD-AA scores more frequently than any other groups, indicating its ability for a consistent improvement. The changes are, however, modest in nature. Although the group PMS has an impressive cumulative ΔRMSD score as shown in Table 3, Figure 5 reveals that changes in RMSD score is not consistent for this predictors. The overall RMSD is improved primarily due to large changes made in three targets (TR671, TR720 and TR722) and not because of consistency. When the best models are considered, MULTICOM-NOVEL has been seen to have notable ability to improve backbone positioning as measured by GDT-TS and RMSD scores by performing a consistent and often large improvement. Apart from i3Drefine, MULTICOM-NOVEL is the only other predictor able to achieve a consistent and simultaneous improvement in ΔGDT-TS and ΔRMSD scores. YASARA group is shown to have promising ability to consistently improve MolProbity score and often with a large degree. In short, if we set aside the difficulty of the predictors to correctly rank their submissions and instead focus on the best structures from each group, we see more successes in refinement.

Overall, i3Drefine method has shown promising ability for a steady improvement in nearly all quality measures both in terms of first or best submitted predictions. The ability of i3Drefine to consistently improve GDT-TS and RMSD scores, which appear to be the most difficult metrics to improve consistently, is also encouraging.

### Head-to-head Comparison of Server Predictors and their Statistical Significance

**Figure 6:**
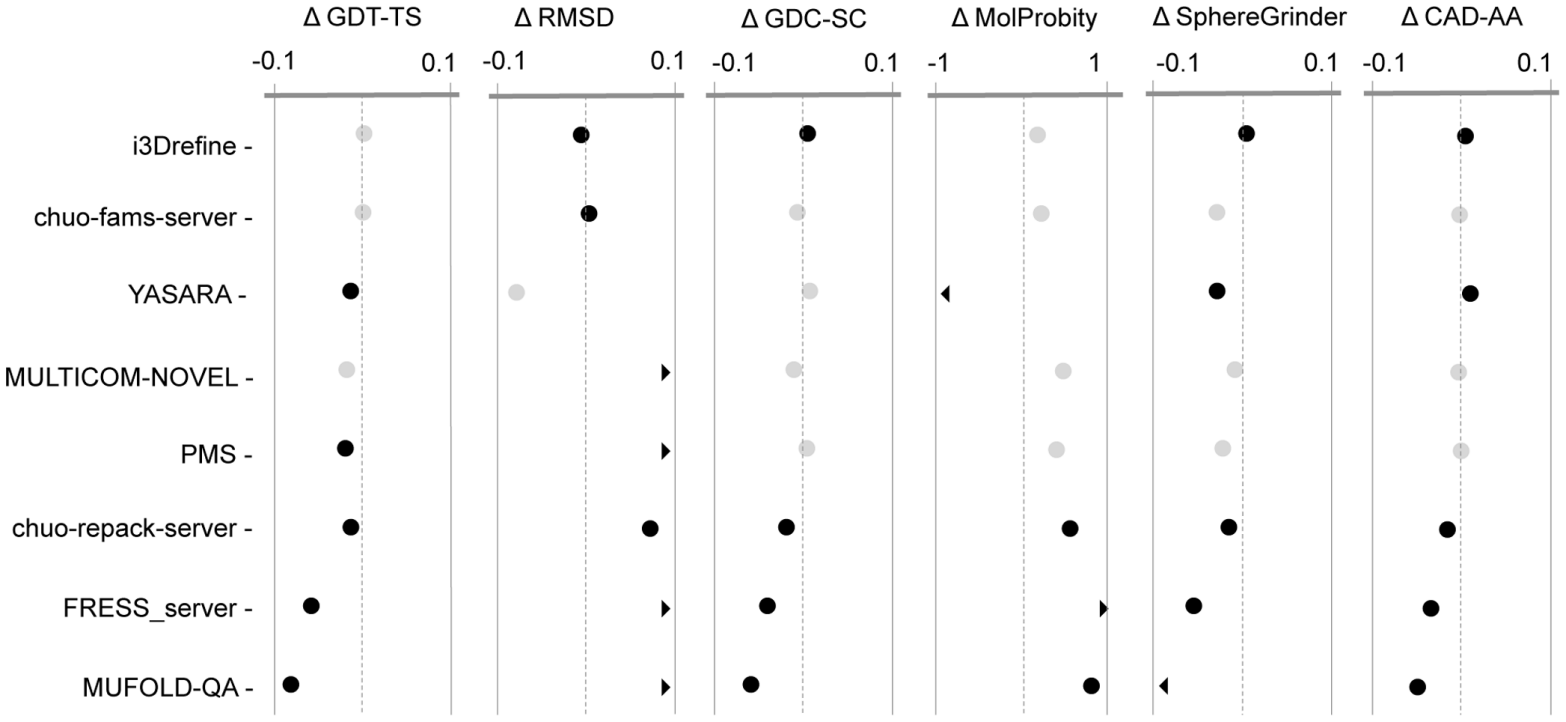
Summary of the average score changes and their statistical significance for top server groups based on best submitted model. Average score changes and their statistical significance relative to starting models for the 8 groups based on the best submitted models as judged by quality score for each target. Each column shows one of the metrics we used to evaluate performance. The scales are marked at ± Average Changes relative to the ‘Void’ group. For GDT-TS, GDC-SC, SphereGrinder and CAD-AA scores, positive changes indicate the quality of the model has been improved by refinement whereas for RMSD and MolProbity, negative changes represent improvement. Black points are statistically distinguishable from the ‘Void’ group; gray points are indistinguishable (Wilcoxon signed-rank test, P = 0.05). A chevron indicates that the corresponding score is off the scale.

**Figure 7:**
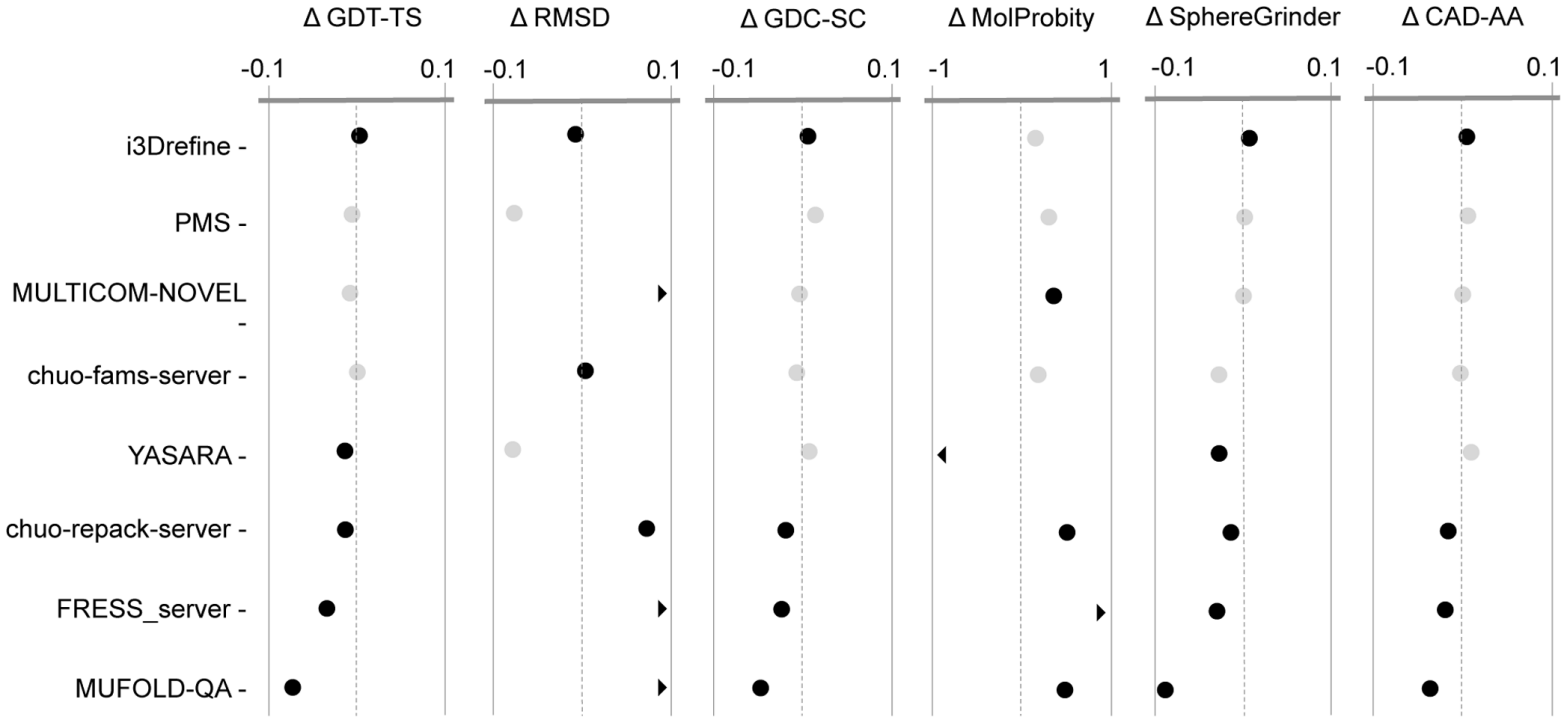
Summary of the average score changes and their statistical significance for top server groups based on best submitted model. Average score changes and their statistical significance relative to starting models for the 8 groups based on the best submitted models as judged by quality score for each target. Each column shows one of the metrics we used to evaluate performance. The scales are marked at ± Average Changes relative to the ‘Void’ group. For GDT-TS, GDC-SC, SphereGrinder and CAD-AA scores, positive changes indicate the quality of the model has been improved by refinement whereas for RMSD and MolProbity, negative changes represent improvement. Black points are statistically distinguishable from the Null group; gray points are indistinguishable (Wilcoxon signed-rank test, P = 0.05). A chevron indicates that the corresponding score is off the scale.

Figure 6 shows the head-to-head comparison in the quality metrics for eight server predictors considering the first model. Upper part of Table 4 summarizes the *p*-values in Wilcoxon signed-rank test with null hypothesis that the refined models are same as the starting structures for eight server predictors. At 5% confidence level, i3Drefine performs statistically significant improvements in RMSD, GDC-SC, MolProbity and CAD-AA scores. The only other group with a statistically significant positive result in for at least one score is YASARA improving MolProbity score significantly. The results remain largely unaffected when judged by the best model for each target. In Figure 7, we present the results for eight server groups considering the overall best models for each target, whose *p*-values of Wilcoxon signed-rank test have been shown in the lower part of Table 4. With the best overall model, i3Drefine performs statistically significant positive result in all quality measures except MolProbity at 5% confidence level. Strikingly, the rest of the server predictors are either indistinguishable from or worse than the ‘Void’ group although the magnitude of average change in scores differs for each method. Given the small number of targets, a group must perform very consistent improvement to be statistically significant with respect to ‘Void’ group and promisingly, i3Drefine is the only server method participating in CASP10 refinement experiment capable to achieve statistically distinguishable improvement in most of the quality metrics.

**Table 4:** *p*-values of score changes (Wilcoxon signed-rank test) relative to starting model for the top server groups in CASP10 refinement experiment.*

Selection	Group Name	P_GDT-TS_	P_RMSD (Å)_	P_GDC-SC_	P_MP_ a	P_SG_ b	P_CAD-AA_
First Model	MULTICOM-CONSTRUCT^c^	0.206377	**0.001270**	**0.000196**	0.102297	**0.035156**	**0.000977**
	chuo-fams-server	0.705321	0.002984	0.420913	0.557197	0.075062	1.000000
	YASARA	0.001715	0.663197	0.338008	**0.000232**	0.029442	0.012940
	MULTICOM-NOVEL	0.101554	0.800817	0.075386	0.107752	0.982396	0.629059
	PMS	0.000590	0.046136	0.782327	0.062602	0.248658	0.930290
	chuo-repack-server	0.000123	0.002466	0.000764	0.000526	0.006444	0.000011
	FRESS_server	0.000024	0.005522	0.000618	6.64*10^−6^	0.004414	0.000017
	MUFOLD-QA	0.000021	0.000392	0.000447	0.001226	0.000214	0.000122
Best Model	MULTICOM-CONSTRUCT^c^	**0.011201**	**0.004240**	**0.000108**	0.178480	**0.004181**	**0.000488**
	PMS	0.312444	0.367622	0.121221	0.107443	0.763939	0.115318
	MULTICOM-NOVEL	0.555264	0.009355	0.485959	0.033486	0.146779	0.803619
	chuo-fams-server	0.705321	0.002984	0.420913	0.557197	0.075062	1.000000
	YASARA	0.001715	0.663197	0.338008	**0.000232**	0.029442	0.012940
	chuo-repack-server	0.000123	0.004268	0.000764	0.000526	0.006444	0.000011
	FRESS_server	0.001396	0.085835	0.014257	7.44*10^−6^	0.015651	0.000402
	MUFOLD-QA	0.000112	0.004061	0.004277	0.027268	0.003859	0.001248

* Numbers in bold indicates statistically significant positive results at P = 0.05.

a P-values for change in MolProbity score.

b P-values for change in SphereGrinder score.

c CASP10 group name for i3Drefine.

Comparison between first model and the best model of i3Drefine shows that effectiveness of the iterative version of the protocol against the non-iterative version (3Drefine). Except MolProbity, the iterative version enhances all the quality measures in terms of cumulative improvement relative to starting models as shown in Table 3. Also, the *p*-values of Wilcoxon signed-rank test are lower for the best model compared to the first model in GDT-TS, RMSD, GDC-SC, MolProbity and CAD-AA scores as reported in Table 4. In short, the degree of refinement as well as their statistical significance in the iterative version is, therefore, more pronounced than the non-iterative version of the protocol.

### Comparison of i3Drefine with Top Five Human Predictors Participating in CASP10

**Figure 8:**
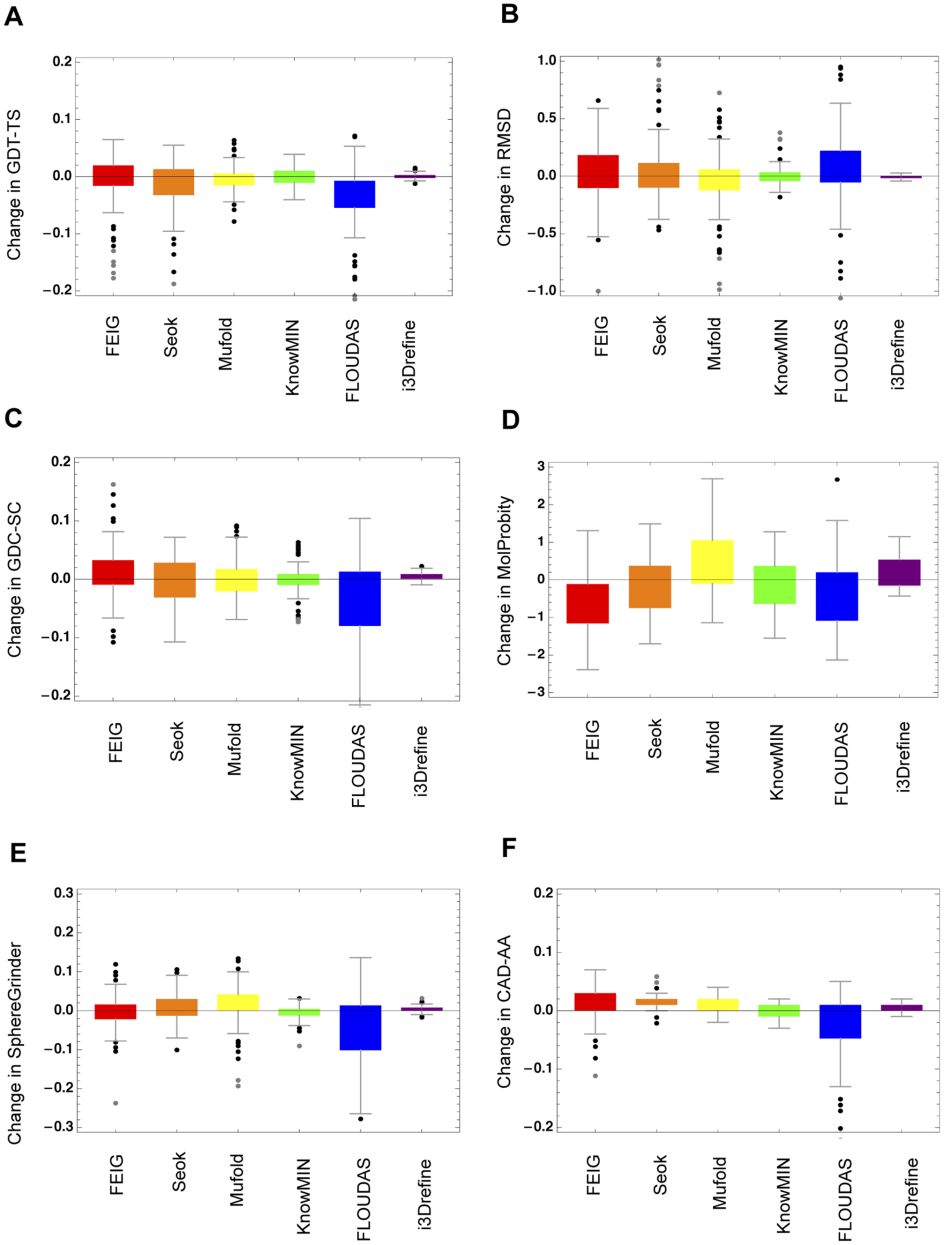
Quartile plots of score changes with respect to the quality of starting structures for top human predictors and i3Drefine. Quartile plots of score changes relative to starting models for 5 human predictors and i3Drefine are shown for these metrics: (A) GDT-TS, (B) RMSD, (C) GDC-SC, (D) MolProbity, (E) SphereGrinder and (F) CAD-AA. The points outside the boxes indicate the outliers.

Figure 8 shows the quartile plots of change in model quality relative to the starting model in six quality metrics for all submitted model by top five human predictors as per the official CASP10 results released during CASP10 meeting and i3Drefine for all CASP10 refinement targets. The most obvious added benefit of human predictors is the ability to perform large improvement in model quality. Groups like FEIG, Seok, Mufold and FLOUDAS seem to perform large changes in starting structures. Although the degree of refinement in these adventurous refinement strategies are much more pronounced than i3Drefine, these methods often lack the ability to perform consistent improvement. Encouragingly, the ability of i3Drefine to perform steady and consistent improvement is noticeable even when it is compared with non-server methods participating in CASP10 refinement experiment. Majority of the times, i3Drefine improves all the quality scores except MolProbity. KnowMIN protocol seems to be more conservative refinement approach than other top-performing human groups. Except SphereGrinder, KnowMIN group improves in the other quality metrics consistently. Among the top-performing human predictors, FEIG group is particularly noteworthy in its ability to improve the backbone positioning as measured by GDT-TS score accompanied by enhancement in local quality measures like MolProbity and CAD-AA. This is possibly achieved through a broader sampling around the starting model. It has to be noted, however, that the human predictors were given three weeks deadline to submit the refined structures to the prediction centre as opposed to three days deadline offered for the server methods and there might be significant human intervention involved in the non-server prediction primarily because of the relaxed submission window. A server group like MULTICOM-CONSTRUCT (i3Drefine), on the other hand, has to be completely automated in order to meet the submission deadline. It is, therefore, unfair to directly compare a server method with human groups especially when the turnaround time for a human predictor is not known. Nevertheless, the ability of human predictors to perform larger improvement can advance the field of protein structure refinement, thereby enhancing the accuracy of contemporary computational protein structure prediction methods provided these methods can be automated providing the prediction within a reasonable amount of time. In addition to being directly implemented in an automated server, human predictors in the CASP experiments often generate valuable insights and guidance for improving protein structure refinement in general.

### Conclusions

In this work, we present a computationally inexpensive and reliable protocol for protein structure refinement, called i3Drefine and systematically analyse its performance in a completely blind mode on the targets issued for refinement category in recently concluded CASP10 experiment based on a diverse set of quality metrics. When compared with other state-of-the-art server predictors participating in CASP10, i3Drefine is observed to perform more consistently than other methods. Future directions would be to explore the possibility of i3Drefine method to perform larger improvement the quality measures by performing a broader sampling around the starting model and possible amendments to the composite force filed. The executable version of i3Drefine software is freely available to the community providing open access to an efficient refinement method. The low computational cost and high accuracy of the i3Drefine protocol will allow this consistent refinement method to be run on a genome scale or be adopted as a final step in computational structure prediction pipeline.
